# Dipeptidyl Peptidase IV Inhibition Activates CREB and Improves Islet Vascularization through VEGF-A/VEGFR-2 Signaling Pathway

**DOI:** 10.1371/journal.pone.0082639

**Published:** 2013-12-11

**Authors:** Balaji Samikannu, Chunguang Chen, Neelam Lingwal, Manju Padmasekar, Felix B. Engel, Thomas Linn

**Affiliations:** 1 Third Medical Clinic and Policlinic, Justus-Liebig-University, Giessen, Germany; 2 CRTD / DFG- Center for Regenerative Therapies Dresden, Paul Langerhans Institut Dresden, Dresden, Germany; 3 University Hospital Erlangen, Experimental Renal and Cardiovascular Research, Nephropathology Division, Department of Pathology, Erlangen, Germany; University of Bremen, Germany

## Abstract

Substitution of pancreatic islets is a potential therapy to treat diabetes and it depends on reconstitution of islet’s capillary network. In this study, we addressed the question whether stabilization of Glucagon-Like-Peptide-1 (GLP-1) by inhibiting Dipeptidyl Peptidase-IV (DPP-IV) increases β-cell mass by modulating vascularization. Mouse or porcine donor islets were implanted under kidney capsule of diabetic mice treated with DPP-IV inhibitor sitagliptin. Grafts were analyzed for insulin production, β-cell proliferation and vascularization. In addition, the effect of sitagliptin on sprouting and Vascular Endothelial Growth Factor (VEGF)-A expression was examined *ex vivo*. The cAMP response element-binding (CREB) and VEGF-A/ Vascular Endothelial Growth Factor Receptor (VEGFR)-2 signaling pathway leading to islet vascularization was explored. Sitagliptin increased mean insulin content of islet grafts and area of insulin-positive tissue as well as β-cell proliferation. Interestingly, sitagliptin treatment also markedly increased endothelial cell proliferation, microvessel density and blood flow. Finally, GLP-1 (7-36) stimulated sprouting and VEGF expression, which was significantly enhanced by sitagliptin- mediated inhibition of DPP-IV. Our *in vivo* data demonstrate that sitagliptin treatment phosphorylated CREB and induced islet vascularization through VEGF-A/VEGFR-2 signaling pathway. This study paves a new pathway for improvement of islet transplantation in treating diabetes mellitus.

## Introduction

Diabetes results from reduction of pancreatic β-cells either by inflammatory processes or by glucolipotoxicity [1]. Elevated levels of circulating fatty acids and glucose contribute to dysfunction of β-cells, leading to type 2 diabetes [2]. Soluble mediators of immune cells, such as oxygen free radicals, nitric oxide, and the cytokines interleukin-1β and interferon- β are thought to induce apoptosis in pancreatic β-cells resulting in type 1 diabetes [3].

It is believed that restoration of deficient β-cell mass is a feasible strategy to treat patients with diabetes mellitus. One approach is transplantation of pancreatic islets. They are endowed with a capillary network that must be reconstituted by *de novo* formation of new blood vessels after transplantation [3], [4]. Despite its requirement the molecular mechanism underlying islet vascularization remains poorly understood.

The key angiogenic molecule VEGF is synthesized in the pancreatic islets, but its expression in isolated islets is significantly reduced [5], [6]. We have previously demonstrated that VEGF increases vascularization of islet grafts [7]. VEGF-A is a secreted mitogen that plays a major role in both angiogenesis and vasculogenesis [8]. Proangiogenic activity of VEGF-A and all the observed vascular endothelial responses are mediated by VEGFR-2 [8]. Similarly the protective role of VEGF-A on neurons and vascular endothelial cells was reported to be due to the phosphorylation of CREB through VEGFR-2/ERK signaling [9].

Interestingly, *in vivo* Glp-1 treatment expands islet mass by increasing pancreatic β-cell number and inducing islet neogenesis in animal models and isolated human islets [10]. The activity of Glp-1 is regulated by DPP-IV that cleaves active Glp-1 (7-36) to Glp-1 (9-36) [11] and mediated by Glp-1R. The cleaved product Glp-1 (9-36) showed mitogenic effects on endothelial cells [12] and promoted vasorelaxation [13]. It has been demonstrated that inhibition of DPP-IV increased Glp-1 availability to the pancreas resulting in increased β-cell mass [14].

Based on the importance of Glp-1 and VEGF-dependent vascularization for the survival of the pancreatic islet graft we hypothesized a novel link between Glp-1, CREB and VEGF-A. Here we show that DPP-IV inhibition by sitagliptin improves vascularization of transplanted pancreatic islets by activation of CREB.

## Materials and Methods

### Animal experiments

Mouse and porcine islets were transplanted into diabetic mice treated with sitagliptin (kindly provided by Dr. Bernd Voss, MSD GmbH, Germany). Animal research was approved by the Regional Commission Giessen, Germany under the code number GI20/11-Nr.15/2006. Animal husbandry was performed according to the German Animal Welfare Law as published in the latest version under http://bundesrecht.juris.de/tierschg.

### Mouse islet transplantation model

We used a murine model of minimal islet mass transplantation. Blood glucose measurement, islet isolation and transplantation were done as described before [7]. Islets isolated from syngeneic donor mice were transplanted under the kidney capsule in C57Bl6 mice made diabetic with streptozotocin (180 mg/kg). Transplantation of about 200 islets was previously determined as optimal cure of diabetes that occurs within 3 days in 90% of recipients. Sitagliptin (5.5% w/w) was administered three days before streptozotocin administration to mice together with their chow (Altromin, Lage, Germany) while control mice received standard lab chow.

### Porcine islet transplantation model

Porcine islets were isolated using previously described techniques of collagenase digestion and Ficoll purification [15], [16] at the Islet Isolation Facility of Third Medical Department, Uni-Giessen. About 2,000 islet equivalents of porcine islets were transplanted beneath the kidney capsule of diabetic nu/nu NMRI mice.

### Insulin content of islet grafts

For insulin measurement, the transplants were explanted, mechanically homogenized and dissolved in acid ethanol, as described before [7]. The supernatant was collected and subjected to species-specific insulin ELISA assays (CV < 6%) after centrifugation (3,000 rpm, 10 min, DRG Instruments GmbH, Marburg, Germany).

### Islet area measurement

Kidneys were retrieved 10 weeks after islet transplantation, snap-frozen in O.C.T medium (Sakura, Netherlands) and serial sections were made (7 µm thick). The sections were fixed in Zamboni’s fixative for 10 min and washed thrice for 5 min in PBS. After blocking in PBS containing 1% BSA and 2% donkey serum, the cryosections were probed with antibodies against insulin (polyclonal guinea pig anti-insulin antibody, Dako, Germany) and mouse monoclonal anti-CD11b antibody M1/70.15 (Immunotools, Germany) at 4°C overnight. CD11b antibody was applied to monitor inflammatory leukocytes elicited by the transplantation procedure. Primary antibodies were visualized with rhodamine-coupled anti-guinea pig and FITC-coupled anti-mouse antibodies (Jackson ImmunoResearch, USA). Samples were mounted with Prolong Gold (Invitrogen), visualized and photographed using a Leica DMLB microscope (Leica, Germany). Insulin-immunostained sections were scanned using Motic image analysis software 2.0 system and analyzed. Total β-cell area was determined in 10 sections from control and sitagliptin-fed group.

### Proliferation of β-cells

Bromodeoxyuridine (Sigma; 100 mg/kg body weight) was injected intravenously for three days to study β-cell proliferation in islet grafts. BrdU double staining with insulin was performed as previously described [7].

### Microvascular density measurements

Microvessels were identified in histologic sections by staining with biotinylated lectin (BS-1, Sigma) as described before [17].

### Blood flow measurements

The blood perfusion of islet graft and adjacent renal cortex was measured by laser-Doppler flow meter (PF 4001-2, Perimed, Stockholm, Sweden) with a needle probe (411 mm tip; outside diameter, 0.45 mm; Perimed). The flow probe was positioned perpendicular to the immobilized tissue surface by the use of a micromanipulator, and care was taken not to cause any compression of the tissue.

### Mouse islet isolation and culture

Pancreatic islets were isolated at the age of 9-12 weeks from C57Bl6 or RIP-VEGF mice, a transgenic mouse model with human vascular endothelial growth factor (VEGF) production in β-cells under the control of the rat insulin promoter (RIP) as described before [7].

### Assessment of VEGF secretion, active Glp-1, and DPP-IV activity

VEGF concentrations of cell culture supernatants and plasma were measured by ELISA (CV< 2%) (R&D Systems, Germany). Plasma Glp-1 Active (7-36) concentrations were measured by ELISA (DRG Instruments GmbH, Germany, CV<3.9%). DPP-IV activity in plasma samples or pancreatic islets was determined using the Glo-reagent for biological fluids (Promega, Mannheim, Germany).

### Ex vivo islet sprouting

Bovine fibrinogen, supplemented with trasylol, was diluted in Dulbecco’s PBS to a final concentration of 1.7 mg/ml. Wells of a 24-well plate were loaded with 300 µl of this solution and all test substances were mixed into the liquid gels. Around 20 to 30 islets were embedded per well after removing the exocrine tissue from 24 h pre-cultured and PBS-washed islets. Gel polymerization was initiated with 5µl of thrombin (1,000 U/ml) at room temperature and gels were subsequently equilibrated with MCDB131 medium (Invitrogen, Germany) containing 5% glutamine in an incubator with 5% CO_2_. The fibrin clots were soaked with 300 µl Parkers FCS medium and cultured for 48 h. Then, gels were fixed with 3% formaldehyde for 6 to 12 h and stained with crystal violet. Washed gels were transferred onto slides and fixed with Kaiser’s glycerol gelatin. Photos were taken and sprout length was determined using Motic software (Wetzlar, Germany).

### Immunocytochemistry and expresssion of VEGF, VEGFR-2, CREB, pCREB, pMTOR and P70S6K

Sections were made from the retrieved graft and fixed with Zamboni’s fixative for 10 min and washed thrice for 5 min in PBS. After blocking in PBS containing 1% BSA and 2% donkey serum, the cryosections were probed with antibodies against insulin (polyclonal guinea pig anti-insulin antibody, Dako, Germany), Rabbit Polyclonal VEGF (Santa Cruz, USA), VEGFR2, CREB and pCREB (Cell Signaling Technology, USA) and Rabbit monoclonal pMTOR and P70S6K antibody (Cell Signaling Technology, USA). Primary antibodies were visualized with FITC- coupled anti-Guinea pig and rhodamine-coupled anti-rabbit antibodies (Jackson Immuno Research, USA). Sections were mounted with Prolong Gold (Invitrogen, Germany), visualized and photographed using Leica DMLB microscope (Leica, Germany). The images were analyzed using Image J software for VEGF and VEGFR-2 area and for number of CREB and pCREB positive β-cell nuclei.

### Statistical analysis

Statistical analysis was performed using Prism 5.0 (GraphPad Software, San Diego California, USA) applying one-way analysis of variance and Bonferroni post-hoc tests. For two-group tests the student *t*-test was applied. Data from the transplantation experiment were plotted as survival curves with ‘cure of diabetes’ as non-recurrent event and analyzed by the Mantel-Haenszel log rank test.

## Results

### Sitagliptin accelerates restoration of normal blood glucose levels

**Figure 1:**
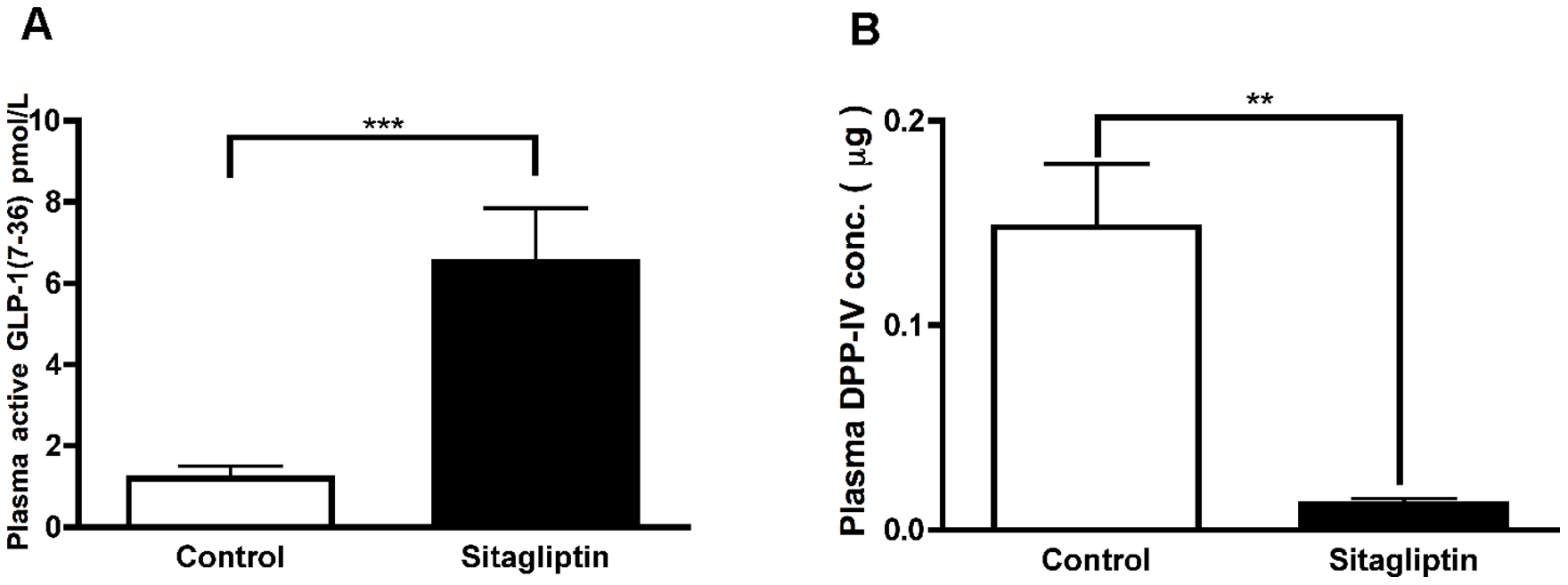
Sitagliptin increases the active GLP-1 level and reduces DPP-IV concentration in plasma. (**A**) Active GLP-1 level in plasma was measured using ELISA kit according to manufacturer’s instructions. Sitagliptin treatment increased the available endogenous active GLP-1 level. (**B**) Plasma concentration of DPP-IV enzyme was measured using Glo-reagent for biological fluids (Promega, Mannheim, Germany) according to manufacturer’s instructions. Sitagliptin treatment inhibited the available DPP-IV enzyme in the plasma. n =  10. Data represents mean ± SEM.

In a previous study DPP-IV inhibition prevented the progression of streptozotocin (STZ)-induced diabetes [18]. To determine the effect of sitagliptin on the cure of diabetes after pancreatic islet transplantation blood glucose levels were measured over a period of ten weeks. Cure of diabetes was defined as sustained reduction of blood glucose < 200 mg/dl. The active Glp-1 level in plasma of mice fed with sitagliptin was 6.5±1.3 pmol/l compared to 1.2±0.3 pmol/l in controls (**Figure 1A**). Plasma DPP-IV activity in control was 0.013 ± 0.002 pmol/min/mg compared to 0.008 ± 0.0006 pmol/min/mg in sitagliptin treated mice (**Figure 1B**).

**Figure 2:**
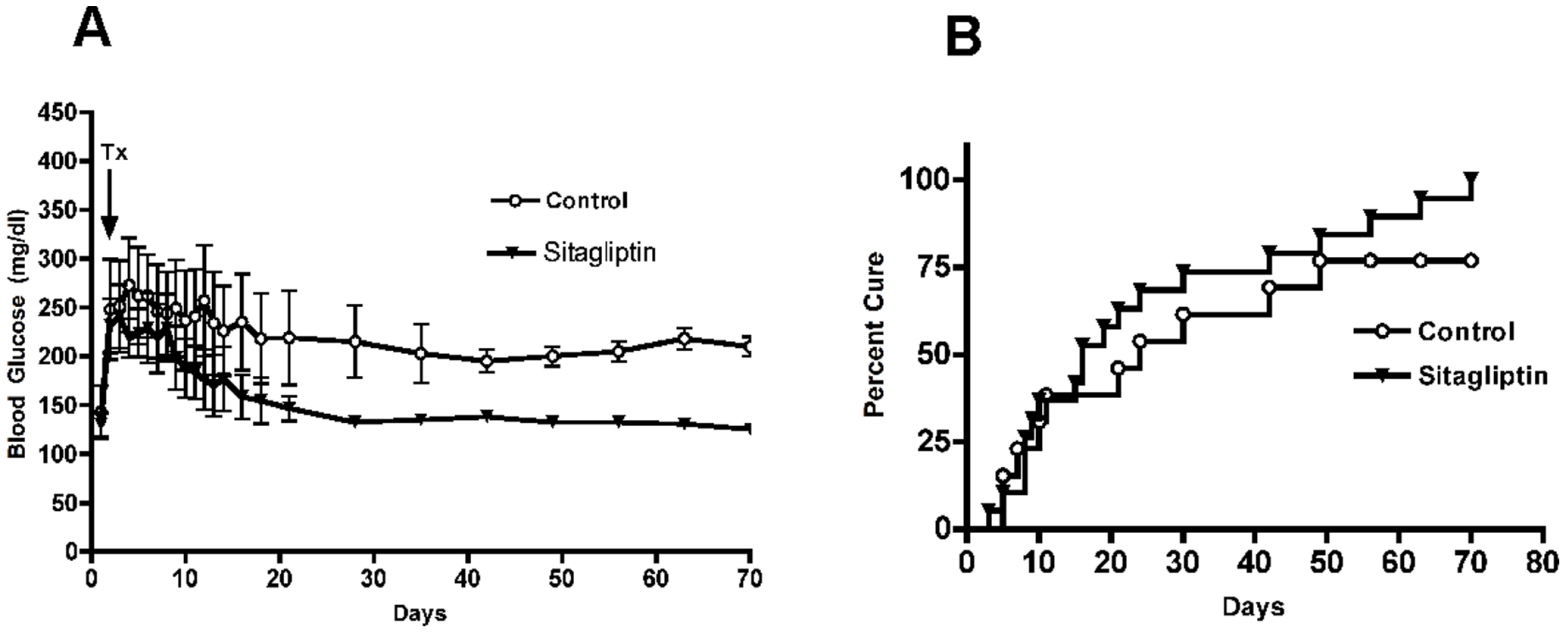
Sitagliptin accelerates restoration of normal blood glucose levels. **(A)** C57Bl6 mice were treated with a single dose of streptozotocin (180 mg/kg) and placed on either a normal chow diet or diet containing 5.5% sitagliptin following syngeneic islet transplantation (day 0). Blood glucose levels (mg/dl) were determined in the control and sitagliptin treated mice. Tx  =  transplantation. Control (White Circles)  =  chow fed mice. Sitagliptin (Inverted Triangle)  =  Recipient mice fed with 5.5% sitagliptin for 10 weeks. (**B**) Graph demonstrating cure from diabetes (< 200 mg/dl) by transplantation up to 70 days after transplantation (p < 0.0072 *vs.* control). n = 12. Data represent mean ± SEM.

Diabetic C57Bl6 mice were hyperglycemic with a mean blood glucose level of 350±80 mg/dl. Subsequent to transplantation with a suboptimal number of murine pancreatic islets blood glucose levels gradually decreased. Three and ten weeks following transplantation 46% and 92% of the control group had returned to non-fasting blood glucose concentrations of less than 200 mg/dl, respectively. In contrast, 100% of sitagliptin-treated mice reached near-normal blood glucose concentrations already after three weeks (**Figure 2** **A, B**). Thus, sitagliptin treatment had a beneficial effect on transplantation-mediated restoration of normal blood glucose levels in diabetic mice.

### Improved insulin production through enhanced β-cell proliferation

**Figure 3:**
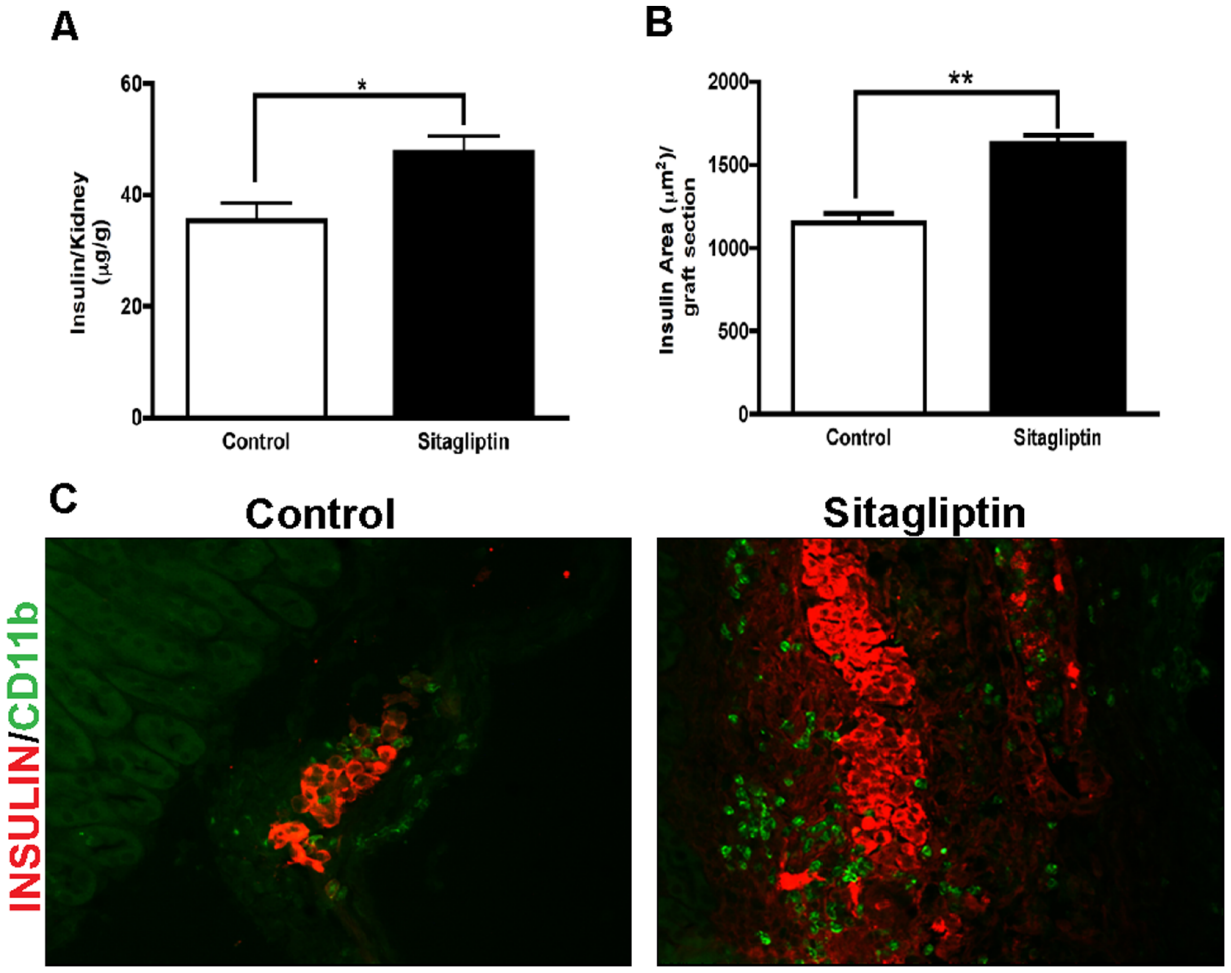
Sitagliptin improves insulin production. (**A**) Insulin content of islet grafts transplanted to the kidney capsule given asµg insulin per g kidney. Control  =  normal chow, Sitagliptin  =  5.5% sitagliptin included into the rodent chow (p < 0.05; n = 6). (**B**) Insulin area (μm^2^) of sections of mouse islet grafts transplanted under the kidney capsule (n = 6, 10 sections per graft, p < 0.01). Data represent mean ± SEM. (**C**) Effect of DDP-IV inhibition by 5.5% sitagliptin on insulin area and immune cell migration to islets transplanted to kidney capsule of NMRI nu/nu mice. Grafted kidneys were recovered as described 10 weeks after transplantation. Representative cryosections were stained for insulin (red) and CD11b (green)-positive immune cells (neutrophils, macrophages). Area stained positive for insulin was larger in the sitagliptin group compared to control. No difference of immune cell infiltration was observed.

To determine the underlying cause of accelerated restoration of blood glucose levels we assessed the effect of sitagliptin on insulin production. Grafts from recipient mice were retrieved and insulin content was determined. The mean insulin content of islet grafts were significantly higher in sitagliptin-treated compared to control (**Figure 3A**). These results were confirmed by immunocytochemistry of sections of transplants utilizing insulin antibodies. Insulin-positive areas were markedly greater in graft sections of sitagliptin-treated mice (**Figure 3B**). Staining with CD11b antibodies revealed no difference of immune cell infiltration (**Figure 3C**).

**Figure 4:**
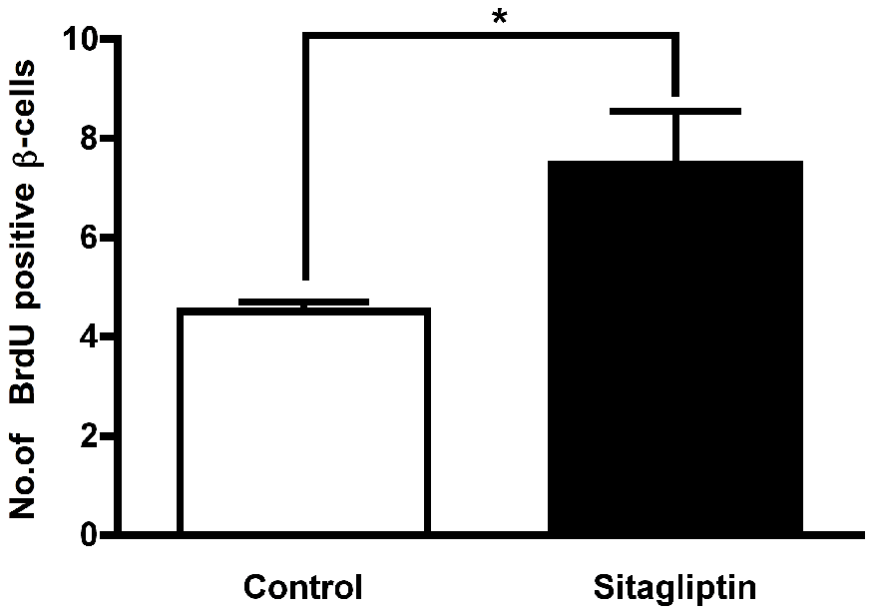
Sitagliptin promotes β*-cell proliferation*. Quantitative analysis of β-cells positive for bromodeoxyuridine (BrdU) in islet grafts transplanted to the kidney capsule of mice on normal chow or chow containing 5.5% sitagliptin (p < 0.05; n = 6; 10 sections per graft). Data represent mean ± SEM.

Larger amounts of insulin can be due to increased insulin biosynthesis per cell or greater number of actively producing β-cells or both. An earlier study reported that sitagliptin treatment in partially pancreatectomized mice increased the β-cell mass in remnant pancreas [19]. Sitagliptin significantly increased the number of BrdU-positive β-cells (**Figure 4**) suggesting that the increased production of insulin after sitagliptin treatment is due to an increased number of β-cells. α- cells were present in the graft, however, no increase in number of α-cells in the sitagliptin group was observed (**Figure S1**).

### Sitagliptin improves vascularization

**Figure 5:**
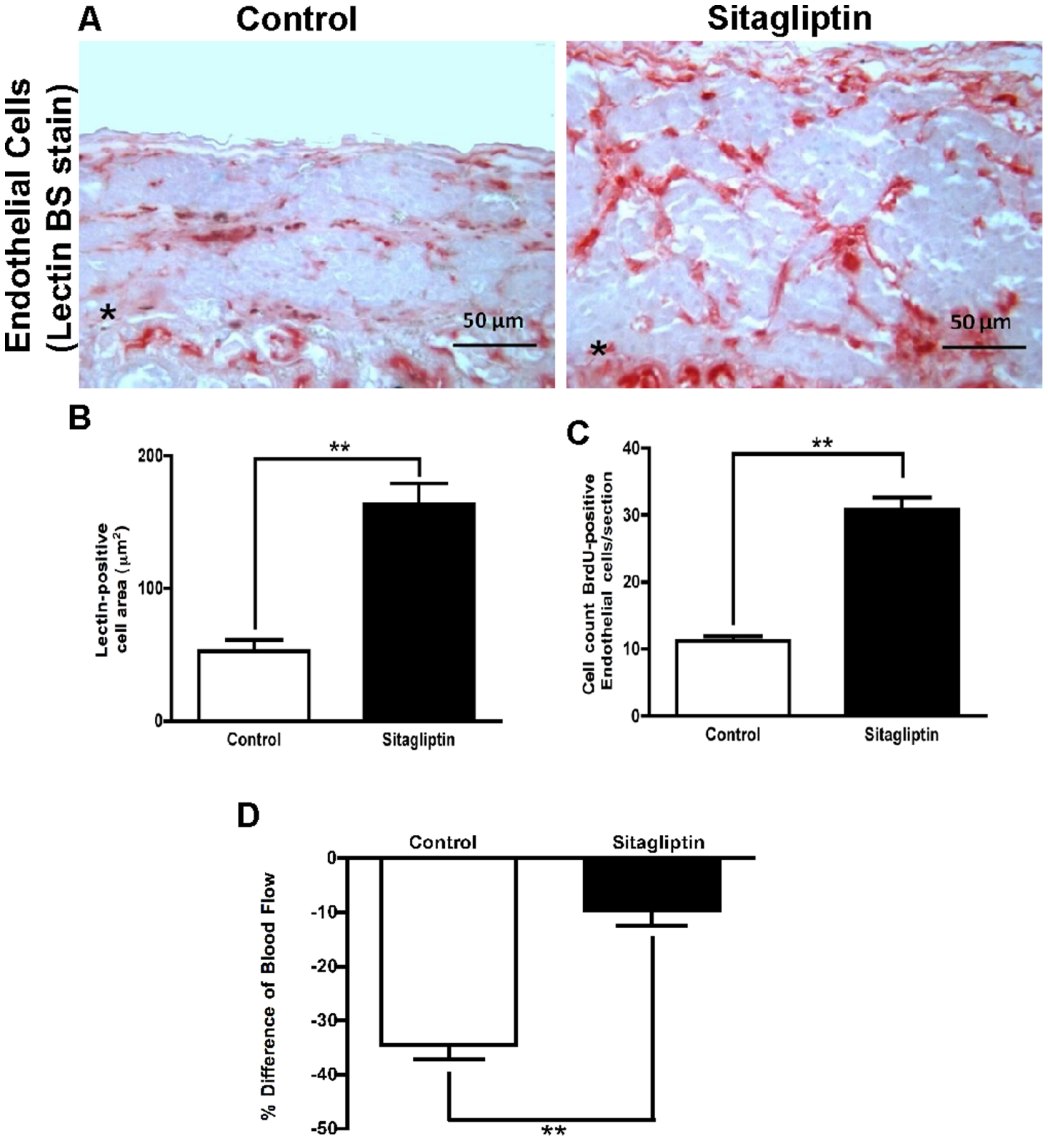
Sitagliptin enhances vascularization and blood flow to the graft. (**A**) Representative picture of immunostaining of islet graft with lectin BS-1 (red) at the kidney capsule of control and sitagliptin-treated islet graft. Both graft area and lectin BS-1-positive areas are larger compared to control. The asterisk * marks the border between islet graft and kidney. (**B**) Quantitative analysis of area (μm^2^) stained positive for lectin BS-1 in islet grafts supplanted to the kidney capsule of C57Bl56 mice fed either with control chow or chow enriched with 5.5% sitagliptin (n = 6; 10 sections per graft, p < 0.01). (**C**) Quantitative analysis of bromodeoxyuridine (BrdU)/lectin BS-1 double-positive cells in islet grafts transplanted to the kidney capsule of mice either on normal chow or chow containing 5.5% sitagliptin (n = 6; 10 sections per graft, p < 0.01). (**D**) Blood flow measured by Doppler ultrasound probe simultaneously on the kidney capsule surface and at the site of the grafted islets. The difference (%) of kidney and graft blood flow is plotted on the ordinate (p < 0.01; n =  6). Data represent mean ± SEM.

Previously, it has been demonstrated that the capillary network of transplanted pancreatic islets has to be reconstituted [7] and vascularization of them occurred within 10–14 days [20]. To examine the effect of sitagliptin, islet transplants were retrieved after 10 weeks. Vascular density within the graft was calculated by morphometry after endothelial cell staining (**Figure 5A**). Sitagliptin treatment significantly increased the area occupied by endothelial cells (**Figure 5B**) as well as the number of proliferating endothelial cells within and around the graft from 11±1 perµm^2^ to 31±5perµm^2^ (**Figure 5C**). We also performed a triple staining to co-localize insulin, glucagon and lectin (BS-1), as the endothelial cell marker. BS-1 positive cells forming microvessels were characteristically observed within and surrounding transplanted islets. Single glucagon positive cells were randomly distributed among the β-cells (**Figure S2**). Collectively, these data suggest that sitagliptin increases proliferation of endothelial cells and vascularization.

### DPP-IV inhibition increases functional blood flow to transplanted islets

To determine if vascularization also resulted in an improved blood flow we applied laser-Doppler flowmetry. A needle probe was used to record signals up to 200 µm under the kidney capsule. Control animals showed a negative difference between the kidney’s and the graft’s regular blood flow. Importantly, the difference in blood flow in sitagliptin-treated mice was significantly lower (**Figure 5D**). These data demonstrate that sitagliptin enhances functional vascularization of transplanted pancreatic islets and improved its viability under transplanted condition.

### Sitagliptin increases VEGF secretion in vitro

**Figure 6:**
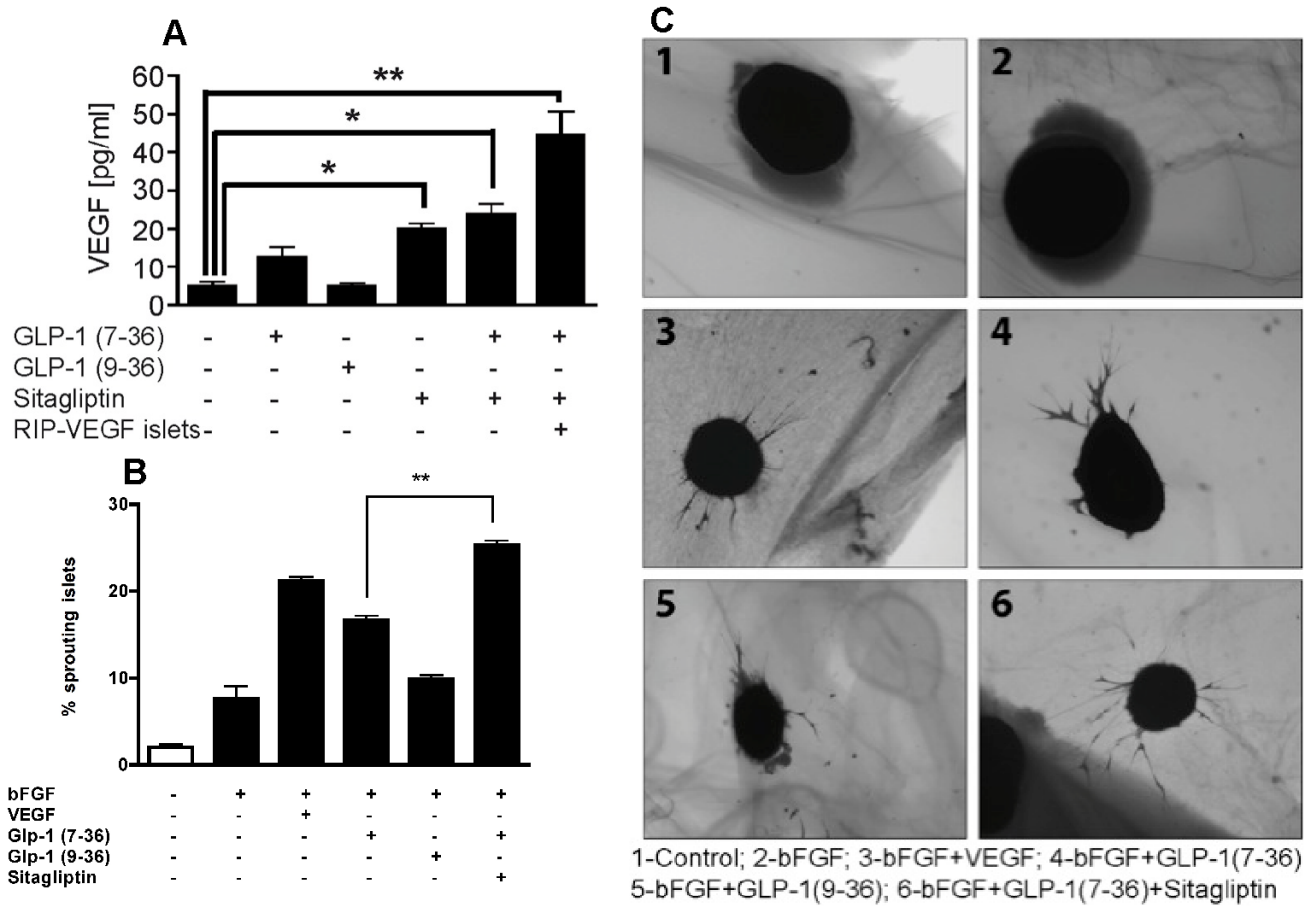
DPP-IV/Glp-1 regulates secretion of VEGF by β-cells. (**A**) Quantitative analysis of VEGF secretion. Mouse islets were cultured free floating in Parkers FCS medium in the presence of Glp-1 (7-36), Glp-1 (9-36), or sitagliptin. RIP-VEGF islets  =  islets isolated from mice synthesizing human VEGFA165 under the control of the rat insulin promoter (RIP). After 24 h of incubation the content of VEGF in the supernatant was determined. Glp-1 (7-36) resulted in higher VEGF content of the supernatant than without the presence of Glp-1 (7-36). Islets cultivated in the presence of Glp-1 (7-36) and 5 µM sitagliptin released even more VEGF (normal islets * p < 0.05 and RIP-VEGF islets ** p < 0.01) compared to islets incubated with Glp-1 (7-36) alone. Data represent mean ± SEM. (**B**) Analysis of the effect of different factors regarding their capacity to induce islet sprouting. bFGF  =  5 ng/ml human basic fibroblast growth factor, VEGF  =  5 ng/ml human vascular endothelial growth factor 165, Glp-1  =  50 nM Glucagon-like peptide 1; either the 7-36 peptide or 50 nM 9-36 fragment. Glp-1 (7-36) is cleaved by dipeptidyl peptidase IV (DPP-IV) resulting in Glp-1 (9-36), sitagliptin  =  5 µM sitagliptin. All data represent mean ± SEM and significance was tested using ANOVA with a Bonferroni post hoc test where ** represents p < 0.01 for the percentage of sprouting islets in the presence of bFGF and Glp-1 (7-36) versus bFGF, Glp-1 (7-36) and DPP-IV inhibitor sitagliptin demonstrating an additional effect on endothelial sprouting by DPP-IV inhibition. (**C**) Representative pictures of sprout-like structures emerging from islets into the fibrin gel due to endothelial proliferation of resident islet endothelial cells.

Local blood perfusion is important for regular function and β-cell survival in islet grafts [3], [4]. Since sitagliptin improved vascularization, we wondered whether it actually increases the VEGF secretion *in vitro* from islets. For this purpose, we treated isolated islets with Glp-1 (7-36) and analyzed the cell culture supernatants for VEGF secretion. Treatment with Glp-1 (7-36) but not Glp-1 (9-36) resulted in a significant increase of VEGF release from normal islets (**Figure 6A**). Sitagliptin further enhanced the effect of Glp-1 (7-36) resulting in a 4-fold increase of VEGF. The use of RIP-VEGF islets demonstrated that VEGF was released from the pancreatic β-cells (**Figure 6A**). These islets were derived from a mouse strain producing VEGF under the control of rat insulin promoter as described before [7]. The result suggests that sitagliptin induces release of VEGF from islets. Next we investigated whether the release of VEGF increases any growth or proliferation of islet endothelial cells *in vitro*.

### Sitagliptin enhances islet endothelial cell sprouting

An earlier study demonstrated that VEGF-A controls angiogenic sprouting in the early postnatal retina [21]. To analyze whether the release of VEGF increases sprouting of islet endothelial cells, we isolated islets and studied the effect of sitagliptin on islet endothelial cell sprouting into fibrin gels. Interestingly, DPP-IV activity was nearly 30-fold higher in islets compared to mouse plasma (6126 ± 320 µmol/min/mg protein *vs.* 215±18 µmol/min/mg, p < 0.0001). The addition of bFGF induced low levels of sprouting, which was greatly enhanced by VEGF. The addition of Glp-1 (7-36) but not addition of Glp-1 (9-36) enhanced the effect of bFGF. As DPP-IV activity was abundant in our assay we tested whether addition of sitagliptin to bFGF + Glp-1 (7-36) can further enhance sprouting. This combination resulted in an even greater sprouting activity as bFGF + VEGF (**Figure 6 B, C)**. Taken together these data suggest that sitagliptin enhances VEGF release and thereby increases islet endothelial cell proliferation necessary for functional viability of transplanted islets by modulating DPP-IV/Glp-1.

Next we worked on the mechanism that promoted VEGF secretion and maintained functional viability of transplanted islets *in vivo.*

### Sitagliptin improves secretion of VEGF in transplanted islets

**Figure 7:**
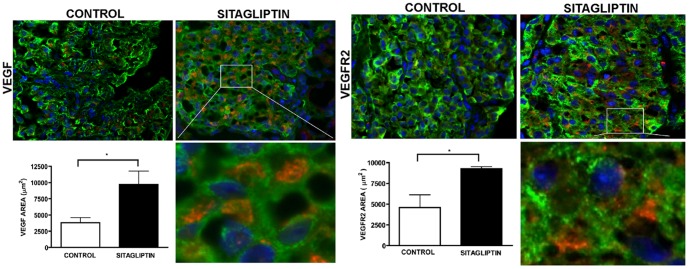
A and B: VEGF signaling pathway is modulated by sitagliptin treatment. Sections were treated with Insulin, VEGF and VEGFR-2 antibodies at 4°C overnight and then treated with secondary antibodies. The image was captured using Leica fluorescent microscope and analyzed using image J software. VEGF and VEGFR-2 staining was more pronounced in the sitagliptin treated group compared to the control. The area showing VEGF and VEGFR-2 staining was significantly (p < 0.05) increased in the sitagliptin group as represented by their respective bar graphs. n  =  4; Data represents mean ± SEM.

**Figure 8:**
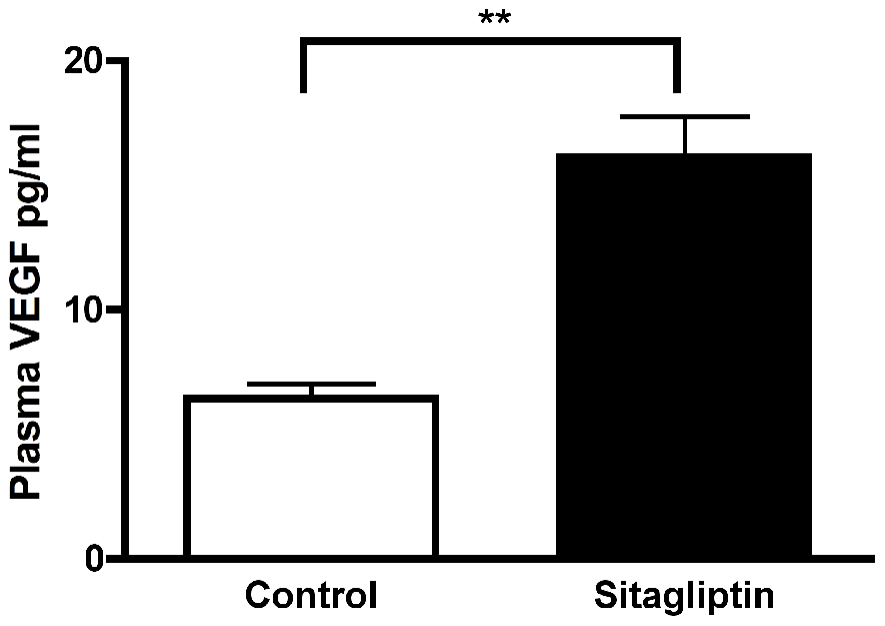
Sitagliptin treatment increases the availability of plasma VEGF level. Plasma VEGF level was measured by using ELISA kit according to manufacturer’s instruction. n = 10; Data represents mean ± SEM.

Since sitagliptin treatment improved transplantation outcome and moreover enhanced VEGF secretion, thereby increasing the sprouting capacity of isolated islets, we examined VEGF secretion from transplanted porcine islets, which will lead to proliferation of host vascular endothelial cells [8]. For this purpose grafts were retrieved and analyzed for the presence of VEGF protein by immunohistochemistry using anti-VEGF antibody. Sitagliptin treatment significantly (p < 0.05) increased the total VEGF expression compared to control (**Figure 7 A**). We also measured the available VEGF in the plasma using ELISA kit and found that sitagliptin treatment increased the VEGF content in plasma (**Figure 8**). VEGF is essential for vasculogenesis- the *de novo* formation of blood vessels [8] and our data indicate that sitagliptin treatment increases the secretion of VEGF improving vasculogenesis.

### Sitagliptin sensitizes VEGFR2 in transplanted islets

VEGF exerts its proangiogenic action by binding to VEGFR-2 [22]. To assess this, graft sections were analyzed for VEGFR2 expression using anti-VEGFR-2 antibody. We found that sitagliptin treatment significantly (p < 0.05) increased VEGFR-2 expression within the transplanted islets (**Figure 7B**). This result indicates that the proangiogenic action of VEGF was further promoted by sensitization of VEGFR-2.

### Sitagliptin activates CREB pathway

**Figure 9:**
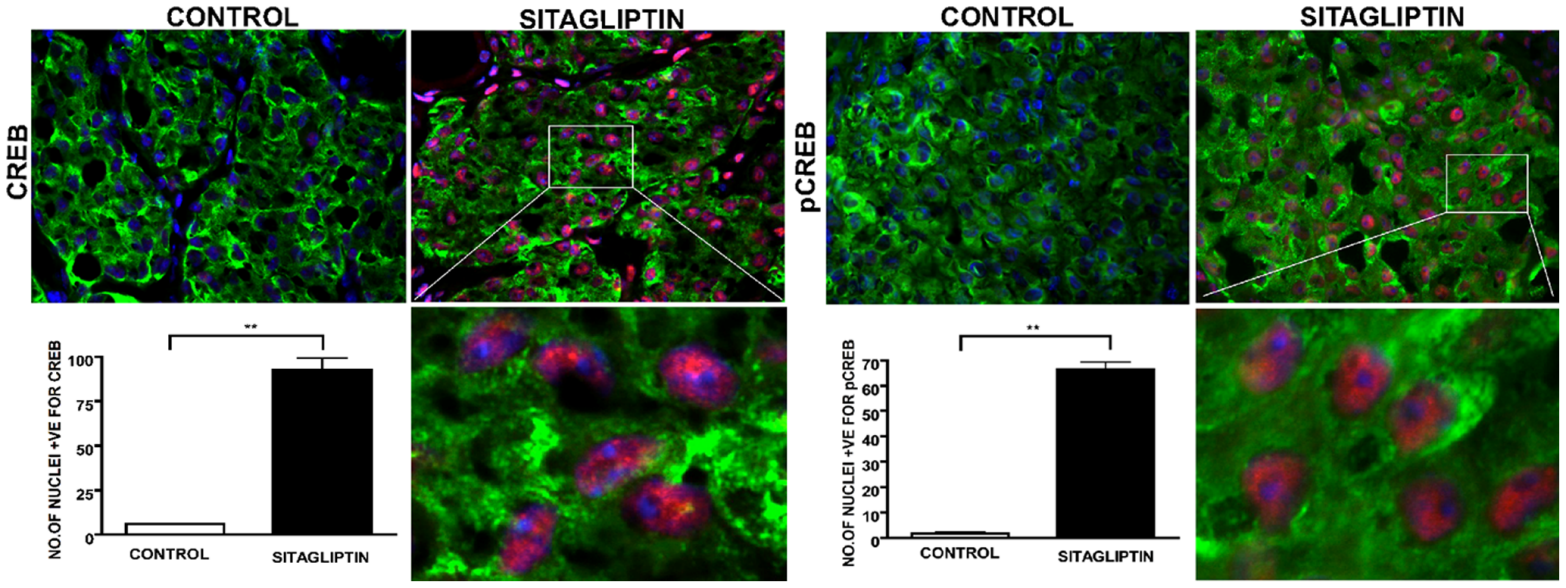
A and B. Sitagliptin phosphorylates CREB and aids in revascularization of islet grafts. Sections were treated with Insulin, CREB and pCREB antibodies at 4°C overnight and then treated with secondary antibodies. The image was captured using Leica fluorescent microscope and analysed using image J software. CREB and pCREB staining was more pronounced in the sitagliptin treated group compared to the control. The number of nuclei stained positive for CREB and pCREB was significantly (p < 0.01) increased in the sitagliptin group as represented by their respective bar graphs. n  =  4; Data represents mean ± SEM.

An earlier study reported that Glp-1 receptor agonists activate CREB and Insulin Receptor Substrate (IRS)-2, thereby promoting β-cell growth and survival [23]. This prompted us to explore whether sitagliptin increases islet vascularization via CREB phosphorylation and VEFG/VEGFR-2 signaling. We retrieved kidney grafts and analyzed them for the activation of CREB by immunocytochemistry. Sitagliptin treatment enhanced CREB expression and phosphorylation compared to control. The number of nuclei in β-cells with translocated CREB protein was significantly (p < 0.01) increased in the sitagliptin-treated group (**Figure 9 A, B**). These results indicate that sitagliptin induces vasculogenesis of transplanted islets by VEGF secretion that would have been affected by phosphorylation of CREB in transplanted β-cells.

### Sitagliptin enhances mTOR and P70S6K expression

**Figure 10:**
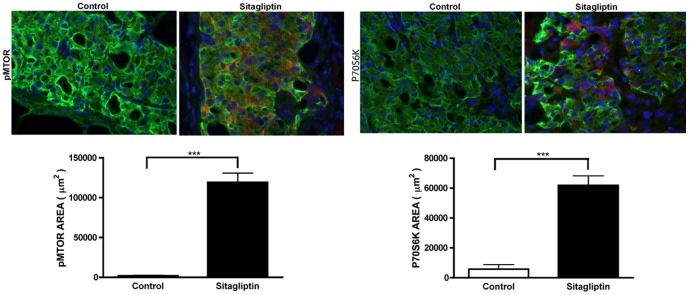
A and B. Sitagliptin phosphorylates mTOR and activates P70S6K. Sections were treated with Insulin, pMTOR and P70S6K antibodies at 4°C overnight and then treated with secondary antibodies. The image was captured using Leica fluorescent microscope. pMTOR and P70S6K staining was significantly (p<0.01) increased in the sitagliptin treated group compared to the control as represented by their respective bar graphs. n  =  4; Data represents mean ± SEM. **C**. Model of CREB, mTOR and P70S6K activation and the VEGF-A/VEGFR-2 pathway regulating secretion of VEGF by DPP-IV inhibition in β-cells.

Since sitagliptin activated CREB in transplanted islets, we next studied the mTOR/P70S6K pathway that will lead to cell growth and proliferation[24]. For this we retrieved kidney grafts and analyzed them for the expression of pMTOR and P70S6K. Sitagliptin treatment increased the expression of pMTOR and P70S6K as evidenced by immunohistochemistry (**Figure 10 A, B**) and pMTOR expression was mainly detected on the pancreatic β-cells (**Figure S3**).

## Discussion

Sitagliptin is considered to enhance active Glp-1 levels by suppressing proteolytic DPP-IV activity resulting in increased β-cell survival in rodent models of diabetes and islet transplantation [18], [25], [26], [27]. On the other hand, it has been demonstrated that the efficiency of pancreatic islet transplantation depends on vascularization [28]. Therefore, we have investigated mechanisms underlying the effect of sitagliptin on pancreatic islet transplantation with focus on angiogenesis. Our data suggest that sitagliptin accelerates normalization of blood glucose level in a diabetes model by stabilizing Glp-1, which in turn results in increased insulin production which may be due to an increase in β-cell mass, VEGF expression and vascularization.

### The DPP-IV-Glp-1-VEGF axis

Pancreatic islets are highly vascularized and formation of their microvessels depends on the expression of VEGF in β-cells [29]. Endothelial cells and microvessels are needed for coordinated insulin secretion of hundreds of β-cells within pancreatic islets [26], [27]. A recent finding indicated that liraglutide, an analog of Glp-1 improved glucose tolerance without affecting islet microcirculation or pancreatic insulin content of native pancreas in Goto Kakizaki rats [30]. Our results show that sitagliptin treatment increased the number of proliferating endothelial cells and also the area occupied by endothelial cells, indicating that actual vascularization takes place after islet transplantation. Studies in mutant mice with pancreas- specific expression of VEGF in pancreatic islets have supported the idea of an association between vascular system and β-cell mass. There is experimental evidence not only in pancreatic islet transplantation [29], [31] but also for STZ-induced diabetes [32]. While endogenous VEGF released from grafted pancreatic islets was beneficial for transplantation outcome, prolonged pharmaceutical administration resulted in decreased islet functionality [33] and microvascular leakage [17]. A recent study suggests that exendin-4 stimulates proliferation of human coronary artery endothelial cells through the eNOS, PKA and PI3K/Akt dependent pathways, which require Glp-1 receptor [12]. However, we cannot confirm this finding for isolated pancreatic islets.

Our data show that DPP-IV activity was detectable in islets isolated with collagenase. Inhibition of DPP-IV by sitagliptin accelerated normalization of blood glucose level in a diabetes model by stabilizing Glp-1[34] and immunostaining of the pancreatic islet grafts showed that Glp1-receptor was almost exclusively expressed on β- cells rather than on endothelial cells (**Figure S4**). This phenomenon correlated with enhanced insulin production due to an increase in β-cell mass. Interestingly, sitagliptin treatment also resulted in an increased expression of VEGF, improved vascularization and functional blood flow to the graft. This correlation *in vivo* raises the question whether vascularization is a direct or indirect effect of sitagliptin treatment. Sitagliptin treatment inhibits DPP-IV, which is known to cleave the hormone Glp-1 (7-36) into its inactive form Glp-1 (9-36). Glp-1 is secreted from the gut and has several functions including the regulation of insulin production [35]. Our finding that VEGF secretion and endothelial cell sprouting is enhanced by inhibition of DPP-IV in the presence of Glp-1 (7-36), but not in the presence of the cleavage product Glp-1(9-36) indicates that DPP-IV regulates VEGF expression via stabilization of Glp-1. Our data furthermore suggest that this axis controls both local and systemic availability of VEGF necessary for vascularization.

The *in vitro* data demonstrated that Glp-1 alone significantly increased VEGF release from pancreatic islets. It is possible that *in vivo* DPP-IV inhibition activated additional pro-angiogenic pathways. There is not only cell membrane abundant peptidase but also a catalytically active soluble form. Basically, the enzyme would cleave dipeptides from all proteins with proline in the penultimate position, however *in vivo* proteolysis could be affected by modulating factors, for example glycosylation of the catalytic site due to hyperglycemia in models of diabetes [36]. DPP-IV is believed to primarily modulate the availability of Glp-1, but other peptides cleaved by DPP-IV, such as Glp-2 or vasoactive peptide [37] could be involved. Chemical inhibition of DPP-IV was reported to reduce attraction of leukocytes in a T-lymphocyte induced immune response [38]. However, recruitment of CD11b+ immune cells into the transplantation site was not different between treated and control in our model.

### Potential intracellular links between Glp-1 receptor activation, CREB phosphorylation, mTOR activation and VEGF synthesis in β-cells

Islet cells are avascular after isolation and reduced vascularity immediately after islet transplantation contributes to the early loss of islets and their function [39]. The vascularization of islets could be increased by several interventions. One approach could be by increasing the proangiogenic factors responsible for formation, proliferation and maturation of islet endothelial cells into fully functional islet vasculature [28]. This approach was demonstrated to increase functional islet mass by improving the angiogenesis of isolated porcine and murine islets [7]. In our study we chose to inhibit DPP-IV so that the available Glp-1 could be used for activation of VEGF, thereby inducing vascularization of transplanted islets. VEGF stimulates mitogenesis of pancreatic ductal epithelium, necessary for β- cell formation [40] and is required for vasculogenesis as well [8]. Our data demonstrate that sitagliptin treatment increased VEGF expression in transplanted islets. Moreover, experimental evidence suggests that the observed vascular endothelial cell response of VEGF was mediated by VEGFR-2 [8]. Our data shows that sitagliptin treatment enhanced the expression of VEGFR-2 in transplanted islets further supporting vascularization.

Interestingly, Glp-1R agonists activate CREB and Insulin Receptor Substrate (IRS)-2, promoting β-cell growth and survival [23]. Also a recent finding suggests that mTOR is linked to the incretin signaling to HIF induction, promoting pancreatic islet viability[41]. Similarly the protective role of VEGF-A on neurons and vascular endothelial cells is by phosphorylation of CREB through VEGFR-2/ERK signaling [9]. Our data show that sitagliptin treatment induced phosphorylation of CREB resulting in its activation of the VEGF-A/VEGFR-2 signaling pathway.

Thus we conclude that the main mechanism of VEGF expression and islet vascularization is Glp-1 stabilization by DPP-IV inhibition through sitagliptin followed by Glp-1R activation leading to phosphorylation of CREB and activation of mTOR, which in turn modulates VEGF-A/VEGFR-2 signaling thereby increasing the vascularization of transplanted islets. The observed increase in proliferative activity of both β-cells and endothelial cells by DPP-IV inhibition may be attributed to activation of P70S6K and that would have been mediated by increased availability of Glp-1 (**Figure 10C**).

From a therapeutic perspective this mechanism could open new venues to prevent the decline of insulin secreting capacity in pancreatic islet transplantation.

## Supporting Information

Figure S1 Pancreatic graft sections were fluorescently stained for Glucagon (Green), proliferative marker Ki67 (Red), and nuclei (DAPI). Merging panels illustrate that α- cells were present in the graft; however, Ki67 stain did not indicate proliferation of α- cells in both the sitagliptin and control group.
(TIF)

Figure S2 Pancreatic islet graft sections were stained for insulin (Blue), glucagon (Green) and endothelial cell marker Lectin (BS-1)(Red). This figure clearly demonstrates that sitagliptin treatment increased the vascularization of islets after transplantation.
(TIF)

Figure S3 Pancreatic islet graft sections were fluorescently stained for insulin (Blue), pMTOR (Green) and endothelial cell marker Lectin (BS-1) (Red). Merging panels show that sitagliptin administration increased pmTOR expression mainly on the pancreatic β- cells.
(TIF)

Figure S4 Sections of pancreatic islet transplants were fluorescently stained for insulin (Blue), GLP-1R (Green) and endothelial cell Lectin (BS-1) (Red). This triple staining clearly demonstrated that Glp1-receptor was almost exclusively expressed on β- cells rather than on endothelial cells.
(TIF)

Methods S1 **Immunohistochemistry and expression of INSULIN, GLUCAGON, GLP-1R, Lectin (BS-1) and Ki67.**
(DOCX)

## References

[1] Prentki M, Joly E, El-Assaad W, Roduit R (2002) Malonyl-CoA signaling, lipid partitioning, and glucolipotoxicity: role in beta-cell adaptation and failure in the etiology of diabetes. Diabetes 51 Suppl 3S405–413. 12475783 10.2337/diabetes.51.2007.s405

[2] McGarry JD, Dobbins RL (1999) Fatty acids, lipotoxicity and insulin secretion. Diabetologia 42: 128–138. 10064091 10.1007/s001250051130

[3] Donath MY, Storling J, Maedler K, Mandrup-Poulsen T (2003) Inflammatory mediators and islet beta-cell failure: a link between type 1 and type 2 diabetes. J Mol Med (Berl) 81: 455–470. 12879149 10.1007/s00109-003-0450-y

[4] Butler AE, Janson J, Bonner-Weir S, Ritzel R, Rizza RA, et al (2003) Beta-cell deficit and increased beta-cell apoptosis in humans with type 2 diabetes. Diabetes 52: 102–110. 12502499 10.2337/diabetes.52.1.102

[5] Zhang N, Richter A, Suriawinata J, Harbaran S, Altomonte J, et al (2004) Elevated vascular endothelial growth factor production in islets improves islet graft vascularization. Diabetes 53: 963–970. 15047611 10.2337/diabetes.53.4.963

[6] Vasir B, Jonas JC, Steil GM, Hollister-Lock J, Hasenkamp W, et al (2001) Gene expression of VEGF and its receptors Flk-1/KDR and Flt-1 in cultured and transplanted rat islets. Transplantation 71: 924–935. 11349728 10.1097/00007890-200104150-00018

[7] Lai Y, Schneider D, Kidszun A, Hauck-Schmalenberger I, Breier G, et al (2005) Vascular endothelial growth factor increases functional beta-cell mass by improvement of angiogenesis of isolated human and murine pancreatic islets. Transplantation 79: 1530–1536. 15940042 10.1097/01.tp.0000163506.40189.65

[8] Robinson CJ, Stringer SE (2001) The splice variants of vascular endothelial growth factor (VEGF) and their receptors. J Cell Sci 114: 853–865. 11181169 10.1242/jcs.114.5.853

[9] Lee HT, Chang YC, Tu YF, Huang CC (2010) CREB activation mediates VEGF-A's protection of neurons and cerebral vascular endothelial cells. J Neurochem 113: 79–91. 20067582 10.1111/j.1471-4159.2010.06584.x

[10] Drucker DJ (2003) Glucagon-like peptides: regulators of cell proliferation, differentiation, and apoptosis. Mol Endocrinol 17: 161–171. 12554744 10.1210/me.2002-0306

[11] Kieffer TJ, McIntosh CH, Pederson RA (1995) Degradation of glucose-dependent insulinotropic polypeptide and truncated glucagon-like peptide 1 in vitro and in vivo by dipeptidyl peptidase IV. Endocrinology 136: 3585–3596. 7628397 10.1210/endo.136.8.7628397

[12] Erdogdu O, Nathanson D, Sjoholm A, Nystrom T, Zhang Q (2010) Exendin-4 stimulates proliferation of human coronary artery endothelial cells through eNOS-, PKA- and PI3K/Akt-dependent pathways and requires GLP-1 receptor. Mol Cell Endocrinol 325: 26–35. 20452396 10.1016/j.mce.2010.04.022

[13] Nathanson D, Erdogdu O, Pernow J, Zhang Q, Nystrom T (2009) Endothelial dysfunction induced by triglycerides is not restored by exenatide in rat conduit arteries ex vivo. Regul Pept 157: 8–13. 19595708 10.1016/j.regpep.2009.07.003

[14] Conarello SL, Li Z, Ronan J, Roy RS, Zhu L, et al (2003) Mice lacking dipeptidyl peptidase IV are protected against obesity and insulin resistance. Proc Natl Acad Sci U S A 100: 6825–6830. 12748388 10.1073/pnas.0631828100 PMC164531

[15] Brandhorst H, Brandhorst D, Brendel MD, Hering BJ, Bretzel RG (1998) Assessment of intracellular insulin content during all steps of human islet isolation procedure. Cell Transplant 7: 489–495. 9786069 10.1177/096368979800700508

[16] Brandhorst H, Brandhorst D, Hering BJ, Bretzel RG (1999) Significant progress in porcine islet mass isolation utilizing liberase HI for enzymatic low-temperature pancreas digestion. Transplantation 68: 355–361. 10459538 10.1097/00007890-199908150-00006

[17] Johansson M, Mattsson G, Andersson A, Jansson L, Carlsson PO (2006) Islet endothelial cells and pancreatic beta-cell proliferation: studies in vitro and during pregnancy in adult rats. Endocrinology 147: 2315–2324. 16439446 10.1210/en.2005-0997

[18] Pospisilik JA, Martin J, Doty T, Ehses JA, Pamir N, et al (2003) Dipeptidyl peptidase IV inhibitor treatment stimulates beta-cell survival and islet neogenesis in streptozotocin-induced diabetic rats. Diabetes 52: 741–750. 12606516 10.2337/diabetes.52.3.741

[19] Kim YS, Oh SH, Park KS, No H, Oh BJ, et al (2011) Improved outcome of islet transplantation in partially pancreatectomized diabetic mice by inhibition of dipeptidyl peptidase-4 with sitagliptin. Pancreas 40: 855–860. 21747318 10.1097/MPA.0b013e318214832d

[20] Menger MD, Jaeger S, Walter P, Feifel G, Hammersen F, et al (1989) Angiogenesis and hemodynamics of microvasculature of transplanted islets of Langerhans. Diabetes 38 Suppl 1199–201. 2463196 10.2337/diab.38.1.s199

[21] Gerhardt H, Golding M, Fruttiger M, Ruhrberg C, Lundkvist A, et al (2003) VEGF guides angiogenic sprouting utilizing endothelial tip cell filopodia. J Cell Biol 161: 1163–1177. 12810700 10.1083/jcb.200302047 PMC2172999

[22] Kinugasa M, Amano H, Satomi-Kobayashi S, Nakayama K, Miyata M, et al.. (2012) Necl-5/Poliovirus Receptor Interacts With VEGFR2 and Regulates VEGF-Induced Angiogenesis. Circ Res. 10.1161/CIRCRESAHA.111.256834 22282193

[23] Jhala US, Canettieri G, Screaton RA, Kulkarni RN, Krajewski S, et al (2003) cAMP promotes pancreatic beta-cell survival via CREB-mediated induction of IRS2. Genes Dev 17: 1575–1580. 12842910 10.1101/gad.1097103 PMC196130

[24] Eguchi M, Masuda H, Kwon S, Shirakura K, Shizuno T, et al (2008) Lesion-targeted thrombopoietin potentiates vasculogenesis by enhancing motility and enlivenment of transplanted endothelial progenitor cells via activation of Akt/mTOR/p70S6kinase signaling pathway. J Mol Cell Cardiol 45: 661–669. 18773906 10.1016/j.yjmcc.2008.08.002

[25] Kim SJ, Nian C, Doudet DJ, McIntosh CH (2008) Inhibition of dipeptidyl peptidase IV with sitagliptin (MK0431) prolongs islet graft survival in streptozotocin-induced diabetic mice. Diabetes 57: 1331–1339. 18299314 10.2337/db07-1639

[26] Toyoda K, Okitsu T, Yamane S, Uonaga T, Liu X, et al (2008) GLP-1 receptor signaling protects pancreatic beta cells in intraportal islet transplant by inhibiting apoptosis. Biochem Biophys Res Commun 367: 793–798. 18211828 10.1016/j.bbrc.2008.01.046

[27] Kirino Y, Sato Y, Kamimoto T, Kawazoe K, Minakuchi K, et al (2009) Interrelationship of dipeptidyl peptidase IV (DPP4) with the development of diabetes, dyslipidaemia and nephropathy: a streptozotocin-induced model using wild-type and DPP4-deficient rats. J Endocrinol 200: 53–61. 18931022 10.1677/JOE-08-0424

[28] Brissova M, Powers AC (2008) Revascularization of transplanted islets: can it be improved? Diabetes 57: 2269–2271. 18753672 10.2337/db08-0814 PMC2518476

[29] Brissova M, Shostak A, Shiota M, Wiebe PO, Poffenberger G, et al (2006) Pancreatic islet production of vascular endothelial growth factor—a is essential for islet vascularization, revascularization, and function. Diabetes 55: 2974–2985. 17065333 10.2337/db06-0690

[30] Wu L, Olverling A, Fransson L, Ortsater H, Kappe C, et al (2012) Early intervention with liraglutide improves glucose tolerance without affecting islet microcirculation in young Goto-Kakizaki rats. Regul Pept 177: 92–96. 22587909 10.1016/j.regpep.2012.05.091

[31] Lammert E, Cleaver O, Melton D (2001) Induction of pancreatic differentiation by signals from blood vessels. Science 294: 564–567. 11577200 10.1126/science.1064344

[32] Linn T, Schneider K, Hammes HP, Preissner KT, Brandhorst H, et al (2003) Angiogenic capacity of endothelial cells in islets of Langerhans. FASEB J 17: 881–883. 12670881 10.1096/fj.02-0615fje

[33] Nakayama S, Uchida T, Choi JB, Fujitani Y, Ogihara T, et al (2009) Impact of whole body irradiation and vascular endothelial growth factor-A on increased beta cell mass after bone marrow transplantation in a mouse model of diabetes induced by streptozotocin. Diabetologia 52: 115–124. 18946656 10.1007/s00125-008-1172-z

[34] Mu J, Petrov A, Eiermann GJ, Woods J, Zhou YP, et al (2009) Inhibition of DPP-4 with sitagliptin improves glycemic control and restores islet cell mass and function in a rodent model of type 2 diabetes. Eur J Pharmacol 623: 148–154. 19765579 10.1016/j.ejphar.2009.09.027

[35] Mortensen K, Christensen LL, Holst JJ, Orskov C (2003) GLP-1 and GIP are colocalized in a subset of endocrine cells in the small intestine. Regul Pept 114: 189–196. 12832109 10.1016/s0167-0115(03)00125-3

[36] Langlois A, Bietiger W, Seyfritz E, Maillard E, Vivot K, et al (2011) Improvement of rat islet viability during transplantation: validation of pharmacological approach to induce VEGF overexpression. Cell Transplant 20: 1333–1342. 21294962 10.3727/096368910X557182

[37] Goke R, Fehmann HC, Linn T, Schmidt H, Krause M, et al (1993) Exendin-4 is a high potency agonist and truncated exendin-(9-39)-amide an antagonist at the glucagon-like peptide 1-(7-36)-amide receptor of insulin-secreting beta-cells. J Biol Chem 268: 19650–19655. 8396143

[38] Aertgeerts K, Ye S, Shi L, Prasad SG, Witmer D, et al (2004) N-linked glycosylation of dipeptidyl peptidase IV (CD26): effects on enzyme activity, homodimer formation, and adenosine deaminase binding. Protein Sci 13: 145–154. 14691230 10.1110/ps.03352504 PMC2286525

[39] Brissova M, Fowler M, Wiebe P, Shostak A, Shiota M, et al (2004) Intraislet endothelial cells contribute to revascularization of transplanted pancreatic islets. Diabetes 53: 1318–1325. 15111502 10.2337/diabetes.53.5.1318

[40] Oberg-Welsh C, Sandler S, Andersson A, Welsh M (1997) Effects of vascular endothelial growth factor on pancreatic duct cell replication and the insulin production of fetal islet-like cell clusters in vitro. Mol Cell Endocrinol 126: 125–132. 9089650 10.1016/s0303-7207(96)03977-9

[41] Van de Velde S, Hogan MF, Montminy M (2011) mTOR links incretin signaling to HIF induction in pancreatic beta cells. Proc Natl Acad Sci U S A 108: 16876–16882. 21949366 10.1073/pnas.1114228108 PMC3193251

