# Supplementary figures and images for: Dipeptidyl Peptidase IV Inhibition Activates CREB and Improves Islet Vascularization through VEGF-A/VEGFR-2 Signaling Pathway
Source: PLoS One. 2013 Dec 11;8(12):e82639. doi: 10.1371/journal.pone.0082639 (PMC3859629; doi:10.1371/journal.pone.0082639)

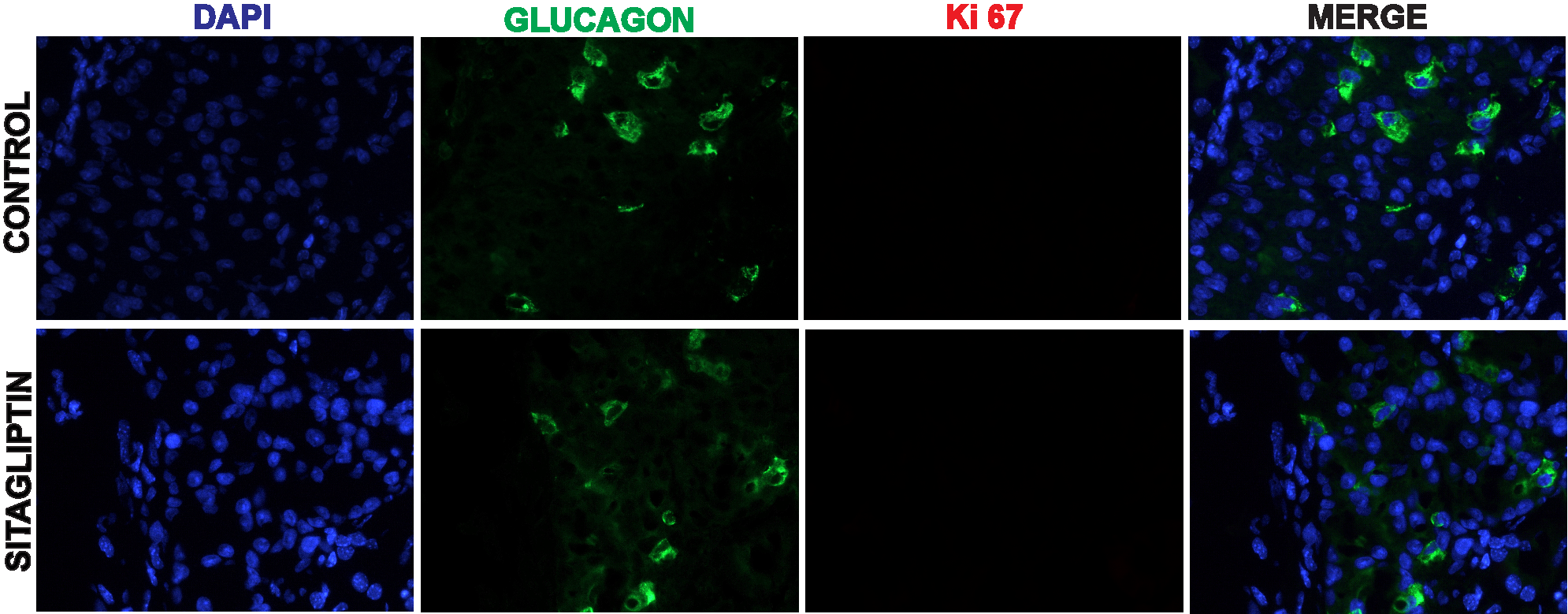

Supplement: Figure S1 — Pancreatic graft sections were fluorescently stained for Glucagon (Green), proliferative marker Ki67 (Red), and nuclei (DAPI). Merging panels illustrate that α- cells were present in the graft; however, Ki67 stain did not indicate proliferation of α- cells in both the sitagliptin and control group. (TIF) [file pone.0082639.s001.tif]

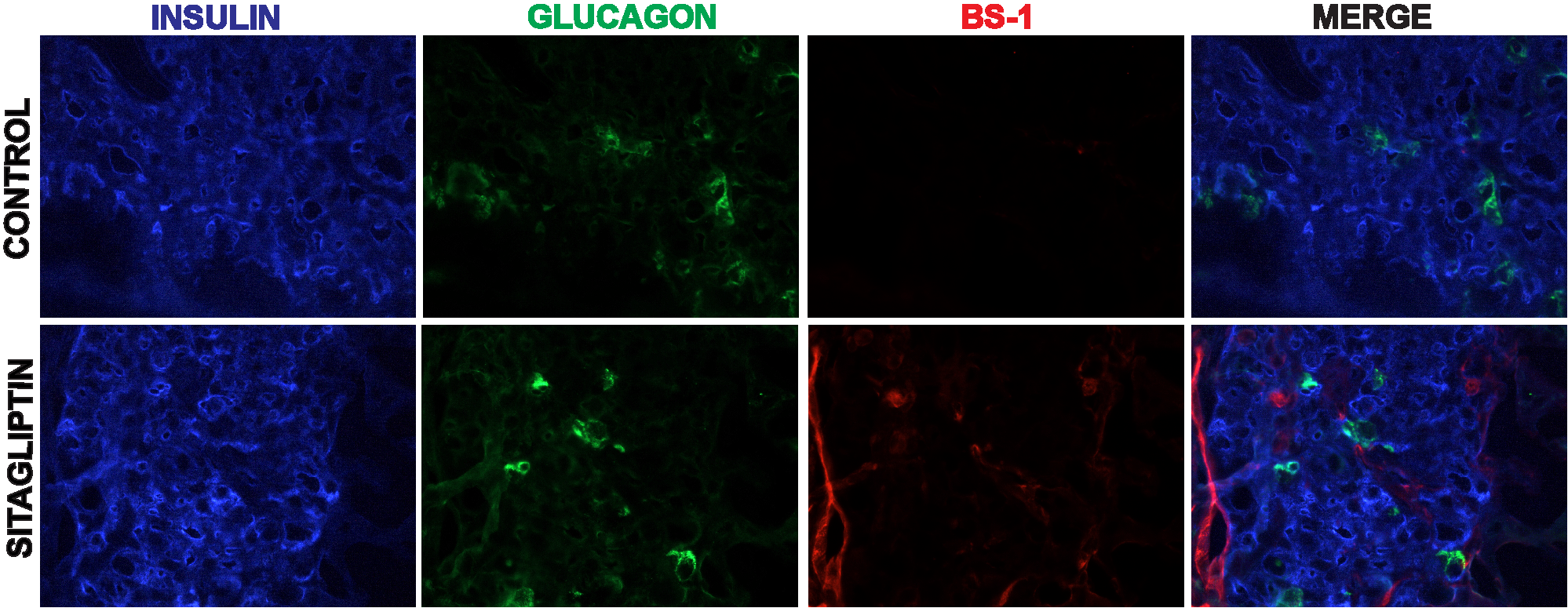

Supplement: Figure S2 — Pancreatic islet graft sections were stained for insulin (Blue), glucagon (Green) and endothelial cell marker Lectin (BS-1)(Red). This figure clearly demonstrates that sitagliptin treatment increased the vascularization of islets after transplantation. (TIF) [file pone.0082639.s002.tif]

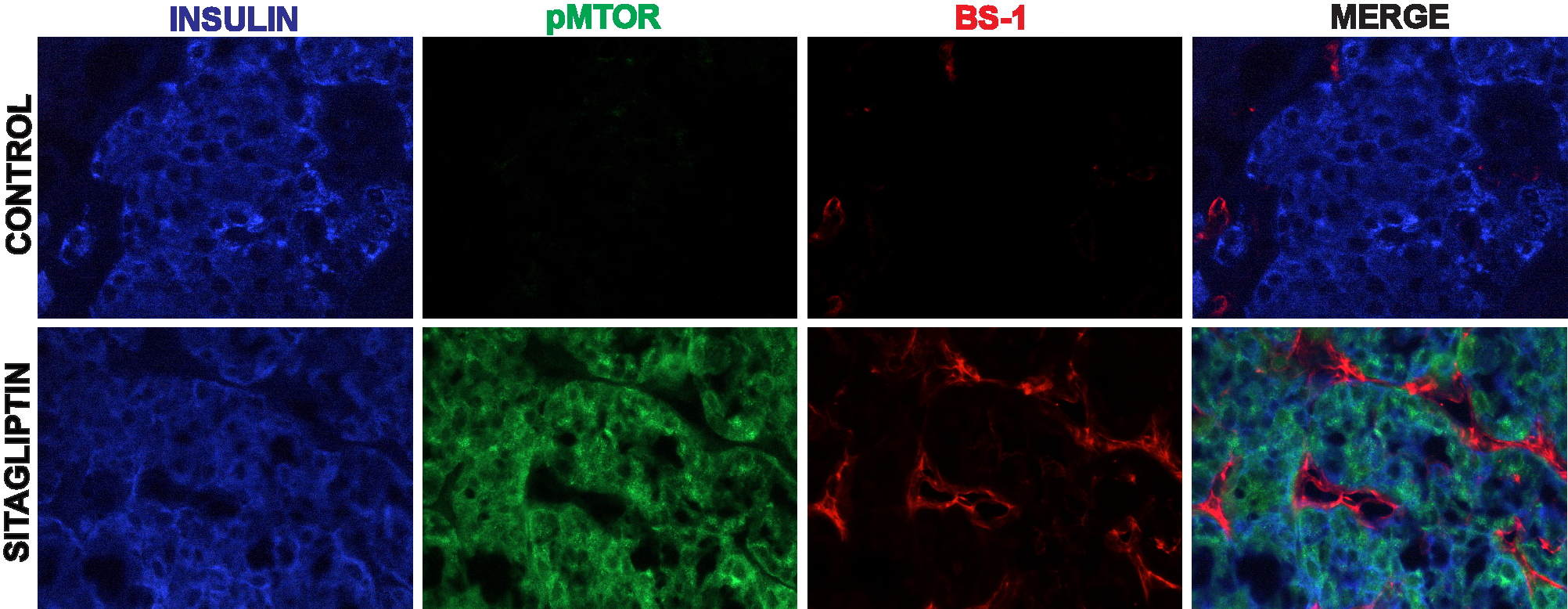

Supplement: Figure S3 — Pancreatic islet graft sections were fluorescently stained for insulin (Blue), pMTOR (Green) and endothelial cell marker Lectin (BS-1) (Red). Merging panels show that sitagliptin administration increased pmTOR expression mainly on the pancreatic β- cells. (TIF) [file pone.0082639.s003.tif]

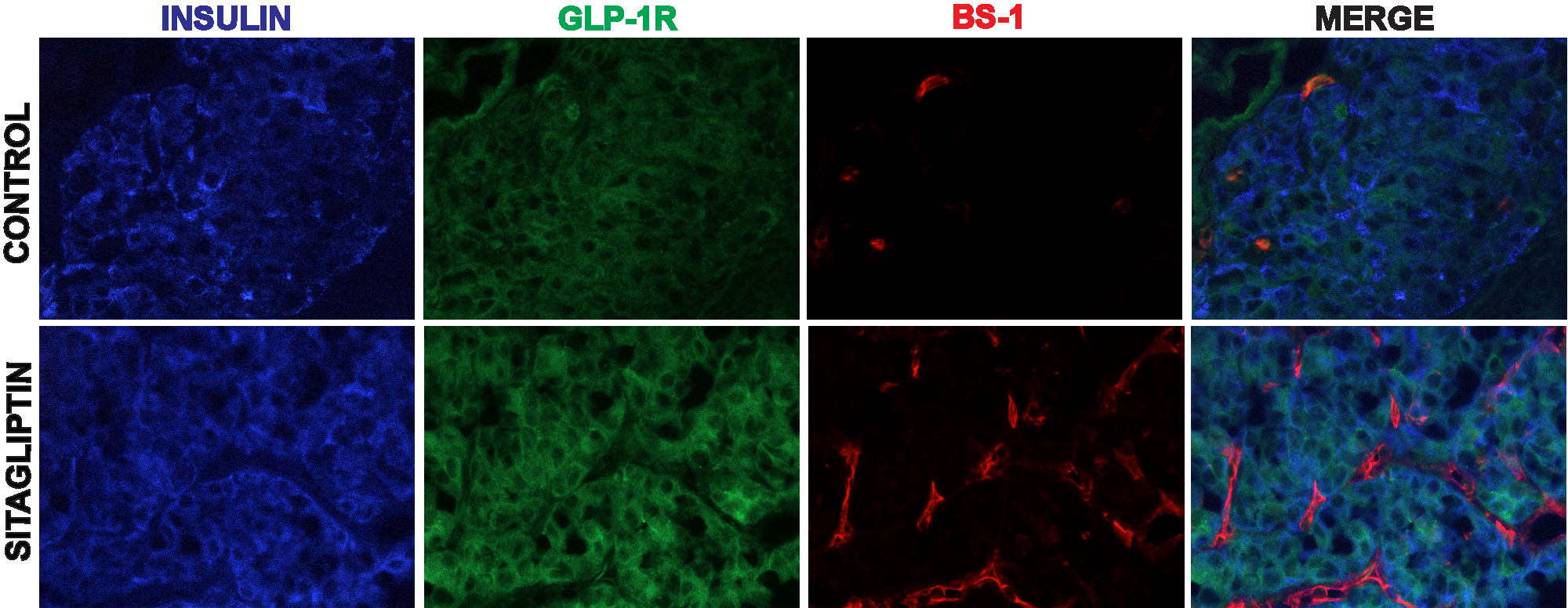

Supplement: Figure S4 — Sections of pancreatic islet transplants were fluorescently stained for insulin (Blue), GLP-1R (Green) and endothelial cell Lectin (BS-1) (Red). This triple staining clearly demonstrated that Glp1-receptor was almost exclusively expressed on β- cells rather than on endothelial cells. (TIF) [file pone.0082639.s004.tif]
