# Supplementary material for: Dipeptidyl Peptidase IV Inhibition Activates CREB and Improves Islet Vascularization through VEGF-A/VEGFR-2 Signaling Pathway
Source: PLoS One. 2013 Dec 11;8(12):e82639. doi: 10.1371/journal.pone.0082639 (PMC3859629; doi:10.1371/journal.pone.0082639)
Supplement: Methods S1 — Immunohistochemistry and expression of INSULIN, GLUCAGON, GLP-1R, Lectin (BS-1) and Ki67. (DOCX) [file pone.0082639.s005.docx]

***Methods S1***

***Immunohistochemistry and expression of INSULIN, GLUCAGON, GLP-1R, Lectin (BS-1) and Ki67***

Sections were made from the retrieved graft and fixed with Zamboni’s fixative for 10 min and washed thrice for 5 min in PBS. After blocking in PBS containing 1% BSA and 2% donkey serum, the cryosections were probed with antibodies against insulin (polyclonal guinea pig anti-insulin antibody, Dako, Germany), Glucagon (polyclonal rabbit anti- Human glucagon, Dako, Germany), GLP-1 Receptor (Rabbit polyclonal to GLP-1R, Abcam, Germany), Ki67 (Monoclonal Rat Anti-mouse Ki67 Antigen Clone TEC-3, Dako, Germany) and Lectin (TRITC conjugated Lectin from Bandeiraea simplicifolia (BS-1), Sigma-Aldrich, Germany) Primary antibodies were visualized with AMCA- coupled Affini pure Donkey anti-Guinea pig, FITC- coupled Affini pure Donkey anti-rabbit and rhodamine-coupled Affini pure Donkey anti-rat antibodies (Jackson Immuno Research, USA). Sections were mounted with Prolong Gold (Invitrogen, Germany), visualized and photographed using Leica DMLB microscope (Leica, Germany).
